# Outcomes after cardiac rehabilitation in patients following repair of thoracic aortic aneurysm or dissection: a protocol for a systematic review and meta-analysis

**DOI:** 10.1186/s13643-023-02180-x

**Published:** 2023-02-07

**Authors:** Niek Koenders, Henrita van Zetten, Michelle Smulders, Martin L. Verra, Roland R. J. van Kimmenade, Thomas van Brakel, Thijs M. H. Eijsvogels, Tim Smith

**Affiliations:** 1 Department of Rehabilitation, Radboud Institute for Health Sciences, Radboud University Medical Center, Nijmegen, the Netherlands; 2 Department of Cardiothoracic Surgery, Radboud University Medical Center, Nijmegen, the Netherlands; 3 Department of Physiotherapy, Inselspital, Bern University Hospital, Berne, Switzerland; 4 Department of Cardiology, Radboud University Medical Center, Nijmegen, the Netherlands; 5 Department of Cardiothoracic Surgery, Catharina Hospital, Eindhoven, the Netherlands; 6 Department of Physiology, Radboud Institute for Health Sciences, Radboud University Medical Center, Nijmegen, the Netherlands

**Keywords:** Thoracic aorta, Dissecting aneurysm, Aortic rupture, Cardiac rehabilitation, Exercise

## Abstract

**Background:**

Patients receiving thoracic aortic repair suffer from long-term impairment in daily functioning and quality of life following intervention due to a combination of their life-threatening condition (i.e. aortic aneurysm or dissection), undergoing major surgery, as well as long-term exercise restrictions thereafter. Despite the known risks of exercise, it is vital that patients regain physical activity in order to recover their daily functioning and quality of life. Cardiac rehabilitation could be a safe and effective treatment to support patients to become physically active by providing exercise training, comprehensive rehabilitation services, and safety recommendations. Despite new insights in recent literature and clinical practice, international guidelines do not recommend cardiac rehabilitation due to limited evidence. We aim to fill this knowledge gap by performing a systematic review and meta-analysis on the effectiveness of cardiac rehabilitation in patients following thoracic aortic repair.

**Methods:**

This protocol has been developed following the Preferred Reporting Items for Systematic Review and Meta-Analysis Protocols (PRISMA-P). MEDLINE, Embase, and CINAHL will be searched for eligible observational and interventional studies from inception up to April 2022. Screening (title/abstract and full text), data extraction, risk of bias assessment, and therapeutic validity rating will be conducted by two independent reviewers. A random-effects model will be used to meta-analyse performance-based outcomes, patient-reported outcomes, clinician-reported outcomes, and researcher-reported outcomes. Subsequently, meta-bias and confidence in evidence will be analysed by two independent reviewers.

**Discussion:**

To exercise or not to exercise in patients following thoracic aortic repair has been a topic of discussion for years. The intended systematic review and meta-analysis will provide comprehensive evidence on the effectiveness of phase III outpatient exercise-based cardiac rehabilitation in patients following thoracic aortic repair. Findings from this review may inform future guidelines for the management of patients with thoracic aortic disease.

**Systematic review registration:**

PROSPERO CRD42022301204.

**Supplementary Information:**

The online version contains supplementary material available at 10.1186/s13643-023-02180-x.

## Background

Thoracic aortic aneurysm or dissection, also known as thoracic aortic disease, is a life-threatening condition [1]. The incidence of thoracic aortic disease is 3–9 per 100,000 people per year [2]. There are surgical techniques to repair thoracic aortic disease via sternotomy, thoracotomy, thoracophrenolaparotomy or endovascular procedures with increasing survival rates in recent decades [3]. However, results following surgery show that patients suffer from long-term impairment in daily functioning and quality of life [4, 5]. Furthermore, there is a risk of aneurysm or dissection in another part of the aorta [6].

Cardiac rehabilitation could be a promising strategy to improve outcomes such as daily functioning and quality of life following thoracic aortic repair. It consists of several core components, including exercise training, lifestyle coaching, medication monitoring to support cardio-vascular risk reduction, healthy behaviour, psychosocial well-being, and an active lifestyle in patients with heart disease [7]. The effects of cardiac rehabilitation are well known for patients with heart failure [8], coronary heart disease [9], and following cardiac surgery [10]. For example, patients with heart failure improved standardised exercise capacity (standardised mean difference 0.98, 95% confidence interval 0.59 to 1.37) [11] and walking capacity (mean 21.0 m; 95% confidence interval 1.57 to 40.4 m) [12] after cardiac rehabilitation compared to controls. Furthermore, patients with coronary artery disease increased health related quality of life (standardised mean change 0.28, 95% confidence interval 0.05 to 0.50) after cardiac rehabilitation [13]. Much less is known about the outcomes of cardiac rehabilitation in patients following thoracic aortic repair.

International guidelines are restraint on cardiac rehabilitation recommendations for patients following thoracic aortic repair. The 2010 ACCF/AHA guideline [14] and 2014 ESC Guidelines on the diagnosis and treatment of aortic diseases [15] do not specify cardiac rehabilitation. Both guidelines recommend to support physically active behaviour and regular aerobic exercise in patients, however, they do not provide treatment strategies for healthcare professionals [14, 15]. The only recommendation is to avoid isometric exercise, strenuous (weight)lifting, pushing and straining (Level III evidence) as this might lead to a superimposed increase in intrathoracic pressure with systolic pressures reaching 300 mmHg that might trigger new aortic rupture [14, 16]. The 2020 ESC guidelines on sports cardiology provide a risk classification and recommends to avoid high and very high intensity exercise, contact, and power-sports [17]. Unfortunately, no recommendations on how to restart other types of exercise are provided. The 2014 Canadian Cardiovascular Society guideline [18] is the first to mention cardiac rehabilitation in patients following thoracic aortic repair as a safe treatment with the potential to reduce mortality. However, they formulated no official treatment recommendation due to a lack of high-quality evidence.

Recent studies indicate that there is an urgent need for cardiac rehabilitation in patients following thoracic aortic repair [4, 5, 19–21]. Patients are challenged by severe exercise restrictions that impair daily functioning and quality of life [5]. An increasing body of evidence questions the need for exercise restrictions and indicate they can do more harm than good [4, 19]. Restrictions result in sedentary behaviour, while being physically active is of great importance for this at-risk patient population [4]. Improving physical activity by cardiac rehabilitation must of course be safe, however, this seems possible by applying safety recommendations such as (1) an early start, (2) tight control of blood pressure, (3) specific training instructions, and (4) strictly avoid competitive sports and isometric training [20]. To our knowledge, there are currently no outcomes known of cardiac rehabilitation in patients following thoracic aortic repair. Although this is important to consider both benefits and risks. Therefore, the main objective of this study is to systematically review current literature and perform a meta-analysis on the outcomes (i.e. daily functioning and quality of life) after cardiac rehabilitation in patients following thoracic aortic repair.

## Methods

### Design

**Fig. 1:**
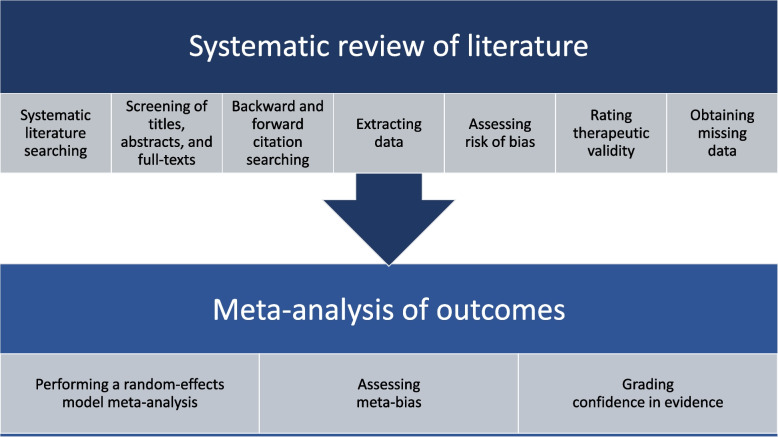
Overview of the study design

Our study protocol follows the Preferred Items for Systematic Reviews and Meta-Analysis – protocol (PRISMA-P) statement [22] and has been registered on PROSPERO CRD42022301204. The systematic review and meta-analysis will be reported in accordance with the PRISMA 2020 statement [23]. The start of the review will be April 2022 and the estimated date for completion is set to December 2022. A summary of the study design is provided in Fig. 1.

### Information sources

A comprehensive electronic search will be conducted in MEDLINE (Ovid), Embase (Ovid), and CINAHL (Cumulative Index to Nursing and Allied Health Literature) Plus (EBSCO) from inception up to April 2022. Additional studies will be identified from (1) reviewing the reference lists of eligible full-text studies through a hand search (backward citation searching) and (2) screening studies that cited the eligible full-text studies according to Web of Science (forward citation searching). Conference proceedings, bibliographies of systematic reviews and trial registers will not be searched. No language or other limitations will be used.

### Search strategy

Observational studies (i.e. cohort studies and registries) and interventional studies (i.e. pilot studies and randomised controlled trials) will be searched for outcomes after cardiac rehabilitation. The search fields ‘Title’, ‘Abstract’, and ‘Medical Subject Headings (MeSH)/Thesaurus’ will be applied to ensure the best possible study retrieval. Detailed search strategies will be developed for each electronic database searched with input from a medical librarian (online supplementary file 1). Pre-searches in MEDLINE to identify search terms and develop a search string were conducted in January-March 2022.

### Eligibility criteria

We will include observational and interventional studies reporting outcomes after phase III cardiac rehabilitation in patients following surgical repair of thoracic aortic aneurysm or dissection. Details of the eligibility criteria are provided in Table 1.

**Table 1:** Details of the eligibility

Patients	- The population will consist of adults 18 years or older following (isolated or combined): aortic root replacement, Bentall/David/Yacoub/Lansac surgery, supracoronary ascending aortic replacement, (partial) aortic arch replacement, (frozen) elephant trunk surgery, or descending thoracic (or thoracoabdominal) aortic replacement - Patients following both open surgery (i.e. sternotomy, thoracotomy, and thoracophrenolaparotomy) and minimally invasive procedures (i.e. hemi-sternotomy) will be included - The population will not consist of adults following isolated aortic valve replacement, endovascular procedures, or conservative treatment
Intervention	- Phase III outpatient exercise-based cardiac rehabilitation - Cardiac rehabilitation is defined as “*exercise training and comprehensive services including education, psychological input, and safety recommendations focusing on health and lifestyle behaviour change, risk factor modification, and psychosocial wellbeing*”[9]
Outcomes	- Performance-based outcomes (e.g. maximal oxygen uptake or maximum walking distance); patient-reported outcomes (e.g. quality of life, fatigue, pain, or perceived exertion); clinician-reported outcomes (e.g. blood pressure or heart rate), and researcher-reported outcomes (e.g. Serious) Adverse events and suspected unexpected serious adverse reactions)
Measurement instruments	- Performance-based measures (e.g. cardiopulmonary exercise test or 6-min walking test); patient-reported measures (e.g. RAND-36, SF-12, numerical rating scale or BORG scale); clinician-reported measures (e.g. blood pressure monitor or electrocardiography), and researcher-reported measures (e.g. study case report forms)
Study types	- Observational studies (e.g. cohort studies) and interventional studies (e.g. randomised controlled trials)
Meta-analysis	- Studies will be included in literature review when it is not clear whether the reported outcomes come only from the patients following thoracic aortic surgery, for example when data from patients following thoracic aortic surgery and cardiac surgery are pooled; however, these outcomes will not be included in the meta-analysis

### Study selection

Retrieved studies will be uploaded to Rayyan software (Rayyan Systems Inc.) [24]. After removing duplicates, two reviewers (NK and HvZ) will independently screen the study against the eligibility criteria. Full text of the (potentially) eligible studies will be thoroughly assessed for inclusion. Reasons for full-text exclusion will be reported (i.e. wrong patient population, wrong outcomes, or wrong intervention). For studies without an available abstract, full-text articles will be obtained unless the article can be confidently excluded by its title alone. Disagreements will be solved by consensus. Where no consensus can be reached, a third party (TS) will arbitrate. In the event of multiple studies reporting findings on the same population, the study with the largest study population will be included. The process of study selection will be summarised using a PRISMA flow diagram [23].

#### Data collection and data management

Data on study characteristics, participant characteristics, and interventions will be extracted to a predefined Excel sheet by two independent reviewers (NK and HvZ). Extracted data will include information on study author(s), year of publication, journal, study design, inclusion criteria, exclusion criteria, number of participants, outcome measures, and follow-up. Details regarding participants in each study will include sex, age, cardiovascular risk factors (i.e. active smoker, alcohol abuse, abdominal circumference, body mass index, diabetes mellitus, dyslipidaemia, family history, and medically treated hypertension), pharmaceutical treatment (i.e. beta blockers), diagnosis received (i.e. aneurysm *versus* dissection), and medical history (i.e. connective tissue disease such as Marfan or Loeys-Dietz). Details of the intervention will be extracted about the rehabilitation program (frequency, intensity, time per session, duration, and type of exercise training [25]; content of education; specifics of psychological input; and safety recommendations) and surgery (type of surgery and level of surgery).

### Outcomes

Data on outcomes will be extracted to the predefined Excel sheet by two reviewers independently (NK and HvZ). Depending on the data reported in the studies, we will collect the raw data, the aggregated outcomes or both. In studies reporting aggregated data, the estimates (i.e. means and medians) will be extracted along with their variation (i.e. 95% confidence intervals, standard error, standard deviation, or range). Details of the outcomes will be extracted about performance-based, patient-reported, clinician-reported, and researcher-reported outcomes (Table 1).

### Risk of bias

The Risk of Bias In Non-randomised Studies of Interventions (ROBINS-I) tool will be used to assess the risk of bias of non-randomised interventional studies [26]. Studies will be independently assessed by two reviewers (NK and HvZ) on confounding, selection bias, information bias, and reporting bias. The Cochrane Risk of Bias Tool 2.0 will be used for randomised interventional studies and independently assessed on the following domains: randomisation process, deviations from intended interventions, missing outcome data, measurement of the outcome, and selection of the reported results [27].

### Therapeutic validity

Therapeutic validity will be scored with the International Consensus on Therapeutic Exercise and Training (i-CONTENT) tool and considers the following seven items: patient selection, dosage of exercise program, type of exercise program, qualified supervisor, type and timing of outcome assessment, safety of exercise program, and adherence to exercise program (online supplementary file 2) [28]. Two reviewers (NK and HvZ) will independently rate the therapeutic validity of the cardiac rehabilitation programmes as either “low risk” or “high risk” of ineffectiveness of the exercise intervention. In case of uncertainty, the item will be evaluated as “probably done” or “probably not done”. Each evaluation will be substantiated with a rationale, which is essentially more important than the low-risk or high-risk score itself.

### Missing data

We will try to contact the original investigators to request missing data. If estimates and variation (i.e. mean and 95% confidence intervals) were reported differently between studies (i.e. median and range), the formula of Hozo et al. [29] will be used to estimate mean and 95% confidence intervals with use of median, range, and sample size. Headrick’s formula [30] will be used to combine means when separate means describe results of one study group. Data from figures will be extracted with the WebPlotDigitizer app (https://automeris.io/WebPlotDigitizer) if not reported in text.

### Data synthesis

If possible, a meta-analysis will be conducted using Review Manager [31]. Extracted data are entered into Review Manager by the first author (NK) and checked by the second author (HvZ). A meta-analysis is possible if outcomes are reported in at least two studies with conceptually the same intervention (i.e. phase III outpatient exercise-based cardiac rehabilitation), outcome domain (e.g. maximal oxygen uptake), and follow-up (e.g. approximately 6 weeks). A random-effects meta-regression model will be used to calculate variance-weighed pooled mean differences and 95% confidence intervals between outcomes before cardiac rehabilitation versus after cardiac rehabilitation. Studies that do not report variance data will be included in the literature review but excluded from the meta-analysis. *I*^2^ statistics are used to assess the heterogeneity, with *I*^2^ statistics ≤ 25% representing low heterogeneity and ≥ 75% (*p* < 0.10) representing high heterogeneity [32]. A subgroup analysis might be performed based on the type of surgery (sternotomy *versus* other) or diagnosis (patients with connective tissue disease *versus* other) [17]. No sensitivity analysis will be performed.

### Meta-bias

For the assessment of meta-bias, outcome reporting bias will be assessed by comparing outcomes listed in the study protocol or methods section with the actually reported outcomes [33]. Publication bias will be visually assessed using a funnel plot [34]. In the presence of publication bias, the funnel plot should resemble an asymmetrical funnel.

### Confidence in evidence

The overall confidence in the body of evidence will be determined with the Grading of Recommendations Assessment, Development, and Evaluation (GRADE) approach [35]. Two reviewers (NK and HvZ) will independently score five categories of reasons for rating down the quality of evidence, and three categories for rating up, with a yes/no decision regarding rating up or down of each outcome. Observational studies will start at a low quality of evidence and can be upgraded accordingly [36]. Studies excluded from the meta-analysis will be excluded from the GRADE assessment. GRADE results will be used to inform conclusions on the overall strength of outcomes after cardiac rehabilitation in patients following repair of thoracic aortic aneurysm or dissection.

## Discussion

A paradigm shift is perceptible in the recent scientific literature. Healthcare professionals are willing to provide cardiac rehabilitation in patients following thoracic aortic repair. However, they do not know what the benefits are, which safety recommendations apply, and whether the risk of adverse events outweighs the benefits [4]. To our knowledge, this will be the first systematic review and meta-analysis that analyses the health benefit of cardiac rehabilitation programmes in patients following thoracic aortic repair. A scoping review was recently published and concluded that the literature is gradually increasing and that the topic is worth studying [37].

There are limitations to this study protocol. First, we excluded studies using endovascular procedures. We acknowledge that thoracic endovascular aortic repair is a promising, safe, and effective procedure to treat descending thoracic aortic aneurysms showing similar perioperative and long-term results compared to open thoracic aortic repair [38]. However, endovascular procedures are not the first-choice solution for ascending aortic repair and aortic arch repair [14]. In addition, some patients are not suitable candidates for endovascular procedures due to an absence of proper ‘landing zones’, too large width of the aorta, lack of vascular access sites, comorbidities, or aetiology [14, 15]. It is important to realise that despite the lower impact of endovascular procedures, cardiac rehabilitation could still be an effective treatment, as has been shown in patients following transcatheter aortic valve implantation [39]. Second, pooled estimates that also include data from patients after isolated cardiac surgery will be excluded from our meta-analysis in order to avoid contamination of the effect-size for patients following thoracic aortic repair.

## Supplementary Information

**Additional file 1. **Concept search strings.

**Additional file 2. **i-CONTENT tool.

## Data Availability

All data generated or analysed during the systematic literature review and meta-analysis will be included in the published article (and its supplementary information files).

## Abbreviations

CINAHL: Cumulative Index to Nursing and Allied Health Literature
MeSH: Medical Subject Headings
PRISMA: Preferred Items for Systematic Reviews and Meta-Analysis
QUIPs: Quality in Prognostic Studies
