# Supplementary material for: Outcomes after cardiac rehabilitation in patients following repair of thoracic aortic aneurysm or dissection: a protocol for a systematic review and meta-analysis
Source: Syst Rev. 2023 Feb 7;12:16. doi: 10.1186/s13643-023-02180-x (PMC9903515; doi:10.1186/s13643-023-02180-x)
Supplement: Supplementary file 1 — Additional file 1. Concept search strings. [file 13643_2023_2180_MOESM1_ESM.docx]

# Supplementary files

## Supplementary file 1 – Concept search strings

MEDLINE search

| Patient terms |
| --- |
| 1. Aorta, Thoracic/ or Thoracic Aortic Aneurysm/ or Aortic Aneurysm/ or Dissecting Aneurysm/ or aortic rupture/ or loeys-dietz syndrome/ or Marfan Syndrome/ 2. (thorac* aort* or aort* root or supracoronary aort* or ascending aort* or aort* arch or partial arch or total arch or descending aort*).ti,ab,kf. 3. ((type ADJ5 dissection*) or (thorac* ADJ5 dissection*) or (type ADJ5 aneurysm*) or (thorac* ADJ5 aneurysm*) or (type ADJ4 aort* rupture) or (thorac* ADJ4 aort* rupture*) or loeys-dietz or marfan*).ti,ab,kf. 4. (thorac* aort* repair or thorac* aort* replace* or thorac* aort* surger* or thorac* aort* operat* or valve-sparing root or Bentall or David or Yacoub or Lansac or elephant trunk).ti,ab,kf. |
| Intervention terms |
| 1. Cardiac Rehabilitation/ or Aneurysm, Dissecting/rh or Aortic Aneurysm, Thoracic/rh or Aortic Aneurysm/rh or Aortic Rupture/rh or Marfan Syndrome/rh or exp Exercise/ or rehabilitation/ or telerehabilitation/ or Physical Therapy Modalities/ or exp exercise movement techniques/ or exp exercise therapy/ or hydrotherapy/ or Respiratory Therapy/ or "Physical Education and Training"/ or health education/ or health promotion/ or exp health behavior/ or patient education as topic/ or smoking prevention/ or exp psychotherapy or relaxation therapy/ or counseling/ or exp directive counseling/ or Distance Counseling/ or Patient Safety/ 2. (rehabilitation OR telerehabilitation OR Exercis* OR training OR physical therapy OR physiotherapy OR kinesiotherapy OR movement therapy OR respiratory therapy OR fitness OR sport* OR Cool* down OR Gymnastics OR Pilates OR Tai Chi OR Yoga OR Plyometric* OR Warm* up OR Running OR Walking OR Swimming OR Stair Climbing OR Jogging OR physical education OR patient education OR psycho-education OR psychoeducation OR health education OR nutrition* education OR health promotion OR (Health* ADJ2 behav*) OR smok* cessation OR psychotherap* OR psychological interventio* OR relaxation therap* OR relaxation techniq* OR counse* OR cognitive therap* OR behav* therap* OR emotion-focused therap* OR psychosocial intervention* OR safety recommendation* OR safety advic* OR safety advis*).ti,ab,kf. |

Embase search

| Patient terms |
| --- |
| 1. Thoracic aorta/ or aortic root/ or ascending aorta/ or aortic arch/ or descending aorta/ or aortic aneurysm/ or exp dissecting aneurysm/ or aortic dissection/ or exp thoracic aorta aneurysm/ or thoracoabdominal aorta aneurysm/ or aortic rupture/ or loeys dietz syndrome/ or marfan syndrome/ or exp thoracic aortic surgery/ 2. (thorac* aort* or aort* root or supracoronary aort* or ascending aort* or aort* arch or partial arch or total arch or descending aort*).ti,ab,kf. 3. ((type ADJ5 dissection*) or (thorac* ADJ5 dissection*) or (type ADJ5 aneurysm*) or (thorac* ADJ5 aneurysm*) or (type ADJ4 aort* rupture) or (thorac* ADJ4 aort* rupture*) or loeys-dietz or marfan*).ti,ab,kf. 4. (thorac* aort* repair or thorac* aort* replace* or thorac* aort* surger* or thorac* aort* operation* or valve-sparing root or Bentall or David or Yacoub or Lansac or elephant trunk).ti,ab,kf. |
| Intervention terms |
| 1. heart rehabilitation/ or exp dissecting aneurysm/rh or exp thoracic aorta aneurysm/rh or aortic aneurysm/rh or thoracoabdominal aorta aneurysm/rh or Marfan syndrome/rh or physiotherapy/ or home physiotherapy/ or exp exercise/ or exp Physical activity/ or rehabilitation/ or telerehabilitation/ or community based rehabilitation/ or functional training/ or home rehabilitation/ or pulmonary rehabilitation/ or respiratory care/ or exp kinesiotherapy/ or exp physical education/ or nutrition education/ or health education/ or health promotion/ or exp health behavior / or patient education/ or psychoeducation/ or smoking prevention/ or smoking cessation program/ or exp psychotherapy/ or counseling/ or directive counseling/ or e-couseling/ or motivational interviewing/ or nutritional counseling/ or patient counseling/ or patient guidance/ or psychological counseling/ or patient safety/ 2. (rehabilitation OR telerehabilitation OR Exercis* OR training OR physical therapy OR physiotherapy OR kinesiotherapy OR movement therapy OR respiratory therapy OR fitness OR sport* OR Cool* down OR Gymnastic* OR Pilates OR Tai Chi OR Yoga OR Plyometric* OR Warm* up OR Running OR Walking OR Swimming OR Stair Climbing OR Jogging OR physical education OR patient education OR psycho-education or psychoeducation OR health education OR nutrition* education OR health promotion OR (Health* ADJ2 behav*) OR smok* cessation OR psychotherap* OR psychological interventio* OR relaxation therap* OR relaxation techniq* OR counse* OR cognitive therap* OR behav* therap* OR emotion-focused therap* OR psychosocial intervention* OR safety recommendation* OR safety advic* OR safety advis*).ti,ab,kf. |

CINAHL search

| Patient terms |
| --- |
| 1. (MH "Aorta, Thoracic") OR (MH "Aortic Aneurysm, Thoracic") OR (MH "Aortic Rupture") OR (MH "Aortic Aneurysm") OR (MH "Aneurysm, Dissecting") OR (MH "Aortic Dissections") OR (MH "Loeys-Dietz Syndrome") OR (MH "Marfan Syndrome") 2. TI (“thorac* aort*” or “aort* root” or “supracoronary aort*” or “ascending aort*” or “aort* arch” or “partial arch” or “total arch” or “descending aort*”) 3. AB (“thorac* aort*” or “aort* root” or “supracoronary aort*” or “ascending aort*” or “aort* arch” or “partial arch” or “total arch” or “descending aort*”) 4. TI ((type N4 dissection*) OR (thorac* N4 dissection*) OR (type N4 aneurysm*) OR (thorac* N4 aneurysm*) OR (type N3 “aort* rupture") OR (thorac* N3 “aort* rupture”) OR "loeys-dietz" OR marfan*) 5. AB ((type N4 dissection*) OR (thorac* N4 dissection*) OR (type N4 aneurysm*) OR (thorac* N4 aneurysm*) OR (type N3 “aort* rupture") OR (thorac* N3 “aort* rupture”) OR "loeys-dietz" OR marfan*) 6. TI ("thorac* aort* repair" OR "thorac* aort* replace*" OR “thorac* aort* surger*” OR “thorac* aort* operation*” OR "valve-sparing root" OR Bentall OR David OR Yacoub or Lansac OR "elephant trunk") 7. AB ("thorac* aort* repair" OR "thorac* aort* replace*" OR “thorac* aort* surger*” OR “thorac* aort* operation*” OR "valve-sparing root" OR Bentall OR David OR Yacoub or Lansac OR "elephant trunk") |
| Intervention terms |
| 1. (MH "Rehabilitation, Cardiac") OR (MH "Aneurysm, Dissecting/RH") OR (MH "Aortic Aneurysm, Thoracic/RH") OR (MH "Aortic Aneurysm/RH") OR (MH "Aortic Rupture/RH") OR (MH "Aortic Aneurysm/RH") OR (MH "Aortic Arch Syndromes/RH") OR (MH "Aortic Dissections/RH") OR (MH "Loeys-Dietz Syndrome/RH") OR (MH "Marfan Syndrome/RH") OR (MH "Exercise+") OR (MH "Physical Therapy+") OR (MH "Rehabilitation") OR (MH "Telerehabilitation") OR (MH "Rehabilitation, Community-Based") OR (MH "Home Rehabilitation+") OR (MH "Rehabilitation, Pulmonary+") OR (MH "Respiratory Therapy") OR (MH "Physical Education and Training") OR (MH "Health Education") OR (MH "Health Promotion") OR (MH "Nutrition Education") OR (MH "Health Behavior") OR (MH "Patient Compliance+") OR (MH "Patient Education") OR (MH "Psychoeducation") OR (MH "Smoking Cessation") OR (MH "Smoking Cessation Programs") OR (MH "Psychotherapy+") OR (MH "Yoga") OR (MH "Tai Chi") OR (MH "Relaxation Techniques") OR (MH "Meditation") OR (MH "Counseling") OR (MH "Nutritional Counseling") OR (MH "Motivational Interviewing") OR (MH "Patient Safety") 2. TI (rehabilitation OR telerehabilitation OR Exercis* OR training OR “physical therapy” OR physiotherapy OR kinesiotherapy OR “movement therapy” OR “respiratory therapy” OR fitness OR sport* OR “cool* down” OR OR Gymnastic* OR Pilates OR "Tai Chi” OR Yoga OR Plyometric* OR “Warm* up” OR Running OR Walking OR Swimming OR “Stair Climbing” OR Jogging OR “physical education” OR “patient education” OR “psycho-education” OR psychoeducation OR “health education” OR “nutrition* education” OR “health promotion” OR (health* N1 behav*) OR “smok* cessation” OR psychotherap* OR “psychological interventio*” OR “relaxation therap*” OR “relaxation techniq*” OR counse* OR “cognitive therap*” OR “behav* therap*” OR “emotion-focused therap*” OR “psychosocial intervention*” OR “safety recommendation*” OR “safety advic*” OR “safety advis*”) 3. AB (rehabilitation OR telerehabilitation OR Exercis* OR training OR “physical therapy” OR physiotherapy OR kinesiotherapy OR “movement therapy” OR “respiratory therapy” OR fitness OR sport* OR “cool* down” OR OR Gymnastic* OR Pilates OR "Tai Chi” OR Yoga OR Plyometric* OR “Warm* up” OR Running OR Walking OR Swimming OR “Stair Climbing” OR Jogging OR “physical education” OR “patient education” OR “psycho-education” OR psychoeducation OR “health education” OR “nutrition* education” OR “health promotion” OR (health* N1 behav*) OR “smok* cessation” OR psychotherap* OR “psychological interventio*” OR “relaxation therap*” OR “relaxation techniq*” OR counse* OR “cognitive therap*” OR “behav* therap*” OR “emotion-focused therap*” OR “psychosocial intervention*” OR “safety recommendation*” OR “safety advic*” OR “safety advis*”) |
