# Supplementary material for: Outcomes after cardiac rehabilitation in patients following repair of thoracic aortic aneurysm or dissection: a protocol for a systematic review and meta-analysis
Source: Syst Rev. 2023 Feb 7;12:16. doi: 10.1186/s13643-023-02180-x (PMC9903515; doi:10.1186/s13643-023-02180-x)
Supplement: Supplementary file 2 — Additional file 2. i-CONTENT tool. [file 13643_2023_2180_MOESM2_ESM.docx]

## Supplementary file 2 – i-CONTENT tool

| 1. Patient selection  Discrepancy between the problems or disabilities of the patient population and the purpose of exercise therapy programme may result in suboptimal effects. |
| --- |
| ‘Low risk’ of ineffectiveness* The purpose of the exercise therapy programme matches the patients’ problems (directly or through a plausible causative relationship). In this case patients’ problems can lie in the International Classification of Functioning, Disability and Health domains of body functions, body structures, and activities and participation. For example:  ►► If the purpose of the exercise programme is to improve a patient’s functional status, then only patients with reduced functional status should be included (direct relationship).  ►► If the purpose of the exercise programme is to improve a patient’s balance, then specifically patients with impaired balance need to be included in the study (direct relationship).  ►► If the purpose of the exercise programme is to improve a patient’s cardiovascular fitness in patients who have been deconditioned as a result of long-term hospitalisation (indirect relationship). |
| ‘High risk’ of ineffectiveness The purpose of the exercise therapy programme does not match the patients’ problems. For example:  ►► If the purpose of the exercise programme is to improve a patient’s quadriceps strength, but patients were not selected on having low quadriceps strength, nor is quadriceps strength a plausible target for the indexed disease |
| 2.Dosage of the exercise programme  The lack of a sound rationale for the dosage of the exercise therapy programme to achieve the purpose of the exercise programme may result in underdosing or overdosing. |
| ‘Low risk’ of ineffectiveness* The investigators applied a plausible or proven rationale based on anatomical, physiological, psychological, neurological, or behavioural relevance to the condition to determine the: Frequency†, Intensity‡, and Time§ of the exercise programme matching  the purpose of the exercise intervention. For example:  ►► Training above 80% of 1RM with low volume for 8 weeks to improve muscle hypertrophy;  ►► Repeating the same movement in high volume to achieve neurophysiological adaptation;  ►► Exercising at least three times a week using resistance training to increase muscular strength. |
| ‘High risk’ of ineffectiveness The investigators did not use a plausible or proven rationale based on anatomical, physiological, psychological, neurological or behavioural relevance to the condition or did not match the purpose of the exercise programme. The investigator did use a plausible or proven rationale based on anatomical, physiological, psychological, neurological or behavioural relevance to the condition, but there is a disconnect between the rationale and the applied Frequency, Intensity, and Time of the exercise programme. For example:  ►► Primarily using cardiovascular exercise to increase muscle hypertrophy.  ►► Primarily exercising above 80% of 1 RM with low frequency to improve stability.  ►► Primarily exercising 1 day a week at 30% HRR to increase muscular strength, using resistance training. |
| 3.Type of the exercise programme  Discrepancy between the type and purpose of the exercise therapy programme may lead to a lack of exercise specificity. |
| ‘Low risk’ of ineffectiveness* The investigators applied a plausible or proven rationale based on anatomical, physiological, psychological, neurological or behavioural relevance to the condition to determine the: Type¶. Furthermore, the investigators matched the Type of the exercise therapy programme with the purpose of the exercise therapy programme. Type of exercise is defined as the form in which the exercise is provided. According to the training specificity principle, it is more likely for training benefits to be transferred to activities if the Type of exercise relates to functional movements. For example:  ►► Cardiovascular exercise through running, cycling or dancing.  ►► Resistance exercise through open/closed chain exercises related to functional targets. Furthermore, if an exercise therapy programme is built up from a no of single exercises, also consider whether there is a plausible or proven rationale for each of the exercises. |
| ‘High risk’ of ineffectiveness The investigators did not match the type of the exercise programme with the purpose of the exercise therapy programme. For example:  ►► Primarily iso-kinetic exercise is prescribed, while the purpose of the programme was walking.  ►► Exercise is primarily delivered by use of exercise bike, while the purpose of the exercise programme was to improve patient-specific activities of daily living. |
| 4.Qualified supervisor (if applicable)  Supervisor(s) who lack the right skills and experiences regarding the exercise programme and patient population may result in suboptimal effects. Note: In case an exercise intervention was not supervised, forgo scoring this item. |
| ‘Low risk’ of ineffectiveness* It can be assumed that the supervisor providing the exercise therapy programme is experienced with the targeted patient population and is sufficiently skilled in providing the proposed exercise programme. For example:  ►► If the content of the evaluated exercise therapy programme falls within the basic skill set of a registered physiotherapist, then the necessary skills can be assumed if the programme is delivered by a physiotherapist. However, the experience with the target population would still need to be made clear.  ►► If the content of the evaluated exercise therapy programme falls outside the basic skill set of a registered physiotherapist, the training and experience in the applied programme need to be defined. |
| ‘High risk’ of ineffectiveness It can be assumed that the supervisor providing the programme is inexperienced with the patient population or is insufficiently skilled to provide the exercise programme. For example:  ►► The exercise programme is provided by a medical specialist without specific training.  ►► The exercise programme is provided to patients aged 25 and below by a medical specialist experienced in geriatric patients. |
| 5.Type and timing of outcome assessment  Using invalid outcome measures or mistiming the measurements might result in the (erroneous) conclusion that an exercise programme was not effective. |
| ‘Low risk’ of ineffectiveness* The investigators used one or more performance-based outcome measures which reflect the goals and purpose of the exercise programme to assess the effectiveness exercise therapy programme. The measurements from the performance-based outcome  measures have taken place within the time window where the expected effect would most likely take place. These performance-based measures need to be valid for the targeted patient population as well as for detecting change over time. For example:  ►► The goal of the exercise programme is to improve functional mobility, and the effect of the exercise programme is measured using the Timed Up and Go test; this test can be assumed to be valid for the targeted patient population and is responsive to measuring change over time. Moreover, the timing of the measurements fall within the period in which the optimal effect can  be expected.** |
| ‘High risk’ of ineffectiveness The investigators use a non-validated (performance) measure as primary outcome measure to assess the effect of the therapeutic intervention. For example, if:  ►► The performance measure applied has not been validated or is unlikely to be valid for the targeted patient group.  ►► The performance measure is known to have a floor or ceiling effect.  ►► The effect of the exercise programme was measured solely by use of self-report measures.  ►► The measurements were obtained outside the window where the optimal effects would be expected. |
| 6. Safety of the exercise programme  A high risk for or no of adverse events may result in a high drop-out rate, reduced adherence and suboptimal effects. |
| ‘Low risk’ of ineffectiveness* The no and severity of the exercise-related adverse events in the study are in line with the expected no of adverse events for similar exercise programmes in similar populations. |
| ‘High risk’ of ineffectiveness The no and severity of the exercise related adverse events are substantially higher than what would be expected, possibly resulting in a higher drop-out  or reduced level of adherence. |
| 7. Adherence to the exercise programme  Low exercise therapy adherence by the patient to the programme may result in a suboptimal effect. |
| ‘Low risk’ of ineffectiveness* Based on relevant information regarding to exercise adherence (ie, the no of sessions attended, the no of exercises performed, and whether or not the intended exercise dosage was reached) the rater draws the conclusion whether the intended exercise dosing was achieved. In order to warrant a ‘low-risk’ conclusion, the level of adherence of patients to the exercise therapy programme is deemed sufficient to assume that the proposed exercise therapy programme was performed as originally intended, in terms of achieved exercise intensity. Cut-off scores may be used to determine whether adherence was deemed adequate, however, we want to stress that the decision needs to be made whether the intended exercise dosing was achieved. |
| ‘High risk’ of ineffectiveness The level of exercise adherence of patients to the exercise therapy programme was insufficient to assume the intended exercise programme was performed as intended. |

*In case insufficient information is provided to judge this item definitively as “low risk”, then it is up to the rater to make a (conservative) judgement as well as provide a rationale for this judgement. †The number of days per week dedicated to the exercise programme. ‡Intensity can be defined using several different measures including but not limited to: percentage of maximal oxygen consumption, oxygen consumption reserve, heart rate reserve, maximal heart rate, or metabolic equivalents. §A measure of amount of time physical activity is performed or by the total caloric expenditure. ¶A variety of exercises to improve the components of physical fitness. **Both the type of the outcome measurement as well as the timing of the outcome measurement should be reasonable to be able to have a “low risk”. If either of the two lacks in either reporting or rationale a high risk of ineffectiveness should be assumed. HRR, heart rate reserve; RM, repetition maximum.
